# Drug-induced hepatotoxicity and association with slow acetylation variants NAT2*5 and NAT2*6 in Cameroonian patients with tuberculosis and HIV co-infection

**DOI:** 10.1186/s12879-024-09638-w

**Published:** 2024-07-31

**Authors:** Frederick Nchang Cho, Eric A. Achidi, Jude Eteneneng Enoh, Srinivas Reddy Pallerla, Le Thi Kieu Linh, Hoang Van Tong, Joseph Kamgno, Véronique Beng Penlap, Ayola Akim Adegnika, Jean-Bernard Lekana-Douki, Marielle Karine Bouyou-Akotet, Gauthier Mesia Kahunu, Gaston Tona Lutete, Mathew Bates, John Tembo, Linzy Elton, Timothy D McHugh, Martin P Grobusch, Alimuddin Zumla, Francine Ntoumi, Thirumalaisamy P. Velavan

**Affiliations:** 1 Institute of Tropical Medicine, University of Tübingen, Wilhelmstrasse 27, 72074 Tübingen, Germany; 2 Faculty of Sciences, University of Buea, Buea, Cameroon; 3 Institute of Medical Research and Medicinal Plants Studies, Yaoundé, Cameroon; 4 Vietnamese-German Centre for Medical Research, VG-CARE, Hanoi, Vietnam; 5 Centre for Research on Filariasis and other Tropical Diseases, Yaoundé, Cameroon; 6 Faculty of Medicine and Biomedical Sciences, University of Yaoundé I, Yaoundé, Cameroon; 7 Department of Biochemistry, Faculty of Sciences, University of Yaoundé 1, Yaoundé, Cameroon; 8 Centre de Recherches Medicales de Lambarene (CERMEL), Lambarene, Gabon; 9 Centre Interdisciplinaire de Recherches Médicales de Franceville (CIRMF), Franceville, Gabon; 10 Department of Parasitology-Mycology and Tropical Medicine, Faculty of Medicine, Université des Sciences de la Santé (USS), Libreville, Gabon; 11 Unit of Clinical Pharmacology and pharmacovigilance, University of Kinshasa, Kinshasa, Democratic Republic of Congo; 12 School of Life Sciences, University of Lincoln, Lincoln, UK; 13 HerpeZ, University Teaching Hospital, Lusaka, Zambia; 14 Centre for Clinical Microbiology, University College London, London, UK; 15 Center for Tropical Medicine and Travel Medicine, Department of Infectious Diseases, Amsterdam University Medical Centers, University of Amsterdam, Amsterdam, The Netherlands; 16 National Institute for Health and Care Research Biomedical Research Centre, University College London, London, UK; 17 Fondation Congolaise pour la Recherche Médicale (FCRM), Brazzaville, Republic of Congo; 18 Faculty of Medicine, Duy Tan University, Da Nang, Vietnam

**Keywords:** Pharmacogenetics, Drug-induced hepatotoxicity, Co-infection, HIV, Tuberculosis

## Abstract

**Background:**

Human immunodeficiency virus (HIV) and tuberculosis (TB) are major contributors to morbidity and mortality in sub-Saharan Africa including Cameroon. Pharmacogenetic variants could serve as predictors of drug-induced hepatotoxicity (DIH), in patients with TB co-infected with HIV. We evaluated the occurrence of DIH and pharmacogenetic variants in Cameroonian patients.

**Methods:**

Treatment-naïve patients with HIV, TB or TB/HIV co-infection were recruited at three hospitals in Cameroon, between September 2018 and November 2019. Appropriate treatment was initiated, and patients followed up for 12 weeks to assess DIH. Pharmacogenetic variants were assessed by allele discrimination TaqMan SNP assays.

**Results:**

Of the 141 treatment naïve patients, the overall incidence of DIH was 38% (53/141). The highest incidence of DIH, 52% (32/61), was observed among HIV patients. Of 32 pharmacogenetic variants, the slow acetylation variants NAT2*5 was associated with a decreased risk of DIH (OR: 0.4; 95%CI: 0.17–0.96; *p* = 0.038), while NAT2*6 was found to be associated with an increased risk of DIH (OR: 4.2; 95%CI: 1.1–15.2; *p* = 0.017) among patients treated for TB. Up to 15 SNPs differed in ≥ 5% of allele frequencies among African populations, while 25 SNPs differed in ≥ 5% of the allele frequencies among non-African populations, respectively.

**Conclusions:**

DIH is an important clinical problem in African patients with TB and HIV. The NAT2*5 and NAT2*6 variants were found to be associated with DIH in the Cameroonian population. Prior screening for the slow acetylation variants NAT2*5 and NAT2*6 may prevent DIH in TB and HIV-coinfected patients.

**Supplementary Information:**

The online version contains supplementary material available at 10.1186/s12879-024-09638-w.

## Introduction

Human immunodeficiency virus (HIV), the causative agent of acquired immunodeficiency syndrome (AIDS), and tuberculosis (TB) are major contributors to morbidity and mortality in sub-Saharan Africa (SSA) [1]. The treatment for HIV and TB mono- or co-infections includes multidrug regimens such as the first-line highly active antiretroviral therapy (HAART) regimen (tenofovir disoproxil fumarate, lamivudine and efavirenz) and RHEZ (rifampicin, isoniazid, ethambutol, pyrazinamide); or HAART plus RHEZ. This often causes drug-drug interactions that cause adverse drug reactions (ADR). Drugs used to treat patients with HIV and TB mono- or co-infections have been reported to cause the highest frequency of adverse events in patients in sub-Saharan Africa such as anorexia, nausea, vomiting, diarrhoea, and hepatotoxicity [2].

Drug-induced hepatotoxicity (DIH) has been reported to occur frequently. DIH can be influenced by various factors including patients’ age, weight, gender, genetic variability, and drug dosages; both at an individual and population level. It is reported that 95% of the differences in the occurrence of DIH are caused by genetic factors alone [3]. Genetic variability of genes involved in the absorption, distribution, metabolism and excretion (ADME) of drugs has been known to influence pharmacokinetics and pharmacodynamics [4]. Pharmacogenetic studies for HIV, TB and malaria drugs have been studied extensively for both ADME and non-ADME gene variants. Some genes that have been studied include *ABCB1*,* CYP2A6*,* CYP2B6*,* CYP3A4*,* CYP3A5*,* CYP2E1*,* GSTMI*,* GSTTI*,* NAT2*,* NR1I3*,* SLCOB1B1*,* UGT2B7*, and *UGT1A9*, which are associated with treatment outcomes and associated toxicities [5]. Particularly, the *NAT2* gene and NAT2*5 and NAT2*6 variants are known to be associated with DIH.

In Cameroon, about half a million people are infected with HIV, and the burden of TB/HIV co-infection is high [6]. The mortality rate of HIV/TB coinfection is high due to lower cure rates [7]. One recent study has indicated a high incidence of DIH of about 41% in patients treated for HIV or TB infections in Cameroon [8]. In addition, there is little information in the Cameroonian population about the genetic predisposition to DIH caused by drugs used to treat TB and HIV infection. Therefore, this study aimed to identify and report on pharmacogenetic variants in the Cameroonian population that are possibly associated with the treatment outcomes of drugs used to treat patients with tuberculosis and HIV infections. In addition, the study assessed genetic predisposition and sought to identify those pharmacogenetic variants that may predict DIH.

## Materials and methods

### Study design and setting

This observational and prospective case-control study was conducted in HIV/AIDS and TB treatment centres of three hospitals: Limbe Regional Hospital (LRH), Buea Regional Hospital (BRH), and Baptist Hospital Mutengene (BHM), in Fako Division, Southwest Region of Cameroon, between September 2018 and November 2019. This study area includes one of the largest HIV/TB treatment centers, which is part of the 223 diagnostic and treatment centers for tuberculosis patients managed by the Cameroon National Tuberculosis Control Program. The BRH and the LRH are central level hospitals. All three hospitals are level 1 hospitals in terms of the volume of HIV tests. All three hospitals are located in the Fako Divisional administrative unit.

### Study participants

For recruitment, individuals reporting to the HIV/AIDS treatment centres; Unité des Pris en Charge (UPEC) of the three study sites for the diagnosis of TB and/ or HIV were adequately sensitised on the study objectives and protocol in order to obtain informed consent. For participants who signed the consent form, we reviewed hospital records for their medical history, to apply our inclusion and exclusion criteria. The inclusion criteria are (1) patients aged ≥ 15 years; (2) newly diagnosed HIV/AIDS patient who accepts to be initiated on HAART; and (3) patients newly diagnosed with TB with positive sputum smear and who accept to be initiated on anti-tuberculosis drug (ATD). A structured questionnaire designed to capture information on socio-demographic characteristics, including gender, age, ethnicity, alcohol abuse, smoking habits, TB and HIV infection treatment regimens, concomitant diseases, and current use of medication was administered to each participant. Participants who had abnormal renal and liver function test results and proven acute/chronic viral hepatitis before the start of treatment were excluded. We also excluded pregnant and lactating women and patients receiving any other hepatotoxic drugs in parallel with HAART and TB drugs from this study. In addition, the patients with interrupted medications (for TB or HIV) were also excluded from this study.

### Treatment, clinical and laboratory investigations

We included HIV, TB or HIV/TB treatment naïve patients in the current study treated with the first-line HAART regimen (Tenofovir Disoproxil Fumarate/Lamivudine/Efavirenz (300/300/400 mg) as recommended. HIV-positive participants, who screened negative for TB, were additionally on cotrimoxazole and isoniazid TB treatment, along with the HAART regimen. About 80% (*n* = 67) of the HIV patients took antimalarial drugs (artemether/lumefantrine) during the follow-up period. Tuberculosis patients received fixed-dose combinations of RHEZ for the first two months, followed by isoniazid and rifampicin for four months. HIV/TB coinfected patients were initiated on regular TB treatment for the first two weeks, after which HAART and cotrimoxazole were added.

A complete history and physical examination were taken before enrolment and at the scheduled visits. After the start of treatment, the patients were followed for 12 weeks. During this time, a morbidity questionnaire was completed before blood sampling at one, four, eight and twelve weeks. During the follow-up visits, DIH and clinical signs such as fever, nausea, vomiting, and fatigue were monitored, and episodes of malaria and opportunistic infections were recorded. The blood was collected and stored at -20 °C until further use, while the serum was stored at -80 °C for further analysis. With the exception of hepatitis B surface antigen and anti-hepatitis C antibody, the following laboratory analyses were performed before and after initiation of treatment; complete blood count (CBC), renal function tests, and liver function tests. The renal function tests were urea and creatinine while the liver function tests included, serum alanine aminotransaminase (ALT), aspartate aminotransaminase (AST), gamma-glutamyl transferase (γ-GT), as well as alkaline phosphatase (ALP), total and direct bilirubin. Follow-up for liver enzymes was performed before treatment, and on the 1st, 4th, 8th, and 12th weeks after initiation of anti-TB treatment or ARV. In addition, for patients whose records lack hepatitis B or C status; a rapid test was carried out to confirm the positivity.

Since DIH is common among patients treated with multiple drugs, we conducted a prospective study based on the WHO criteria (ALT > 1.25 x ULN) to examine the prevalence and incidence of DIH. Serum ALT levels were utilized for grading DIH, and the values were expressed as multiples of the upper limit of the normal range (ULN). The normal ALT values were set as < 32 U/I for females and < 40 U/I for males [9]. The grading of DIH was based on ALT levels as follows: Grade I (mild) 1.25-2.5x ULN; Grade II (moderate) 2.6-3.5x ULN; Grade III (severe) 3.6-5x ULN and Grade IV (life-threatening) > 5x ULN [10].

### Single nucleotide polymorphisms (SNPs) inclusion criteria

SNPs with functional and clinical significance for drugs used to treat HIV infection and TB were selected from the Pharmacogenomics Knowledge Base (PharmGKB; www.pharmgkb.org). SNPs with allele frequencies > 10% known to occur in African populations based on the 1000 Genomes Project [11] were considered. A custom TaqMan™ SNP panel including 32 SNPs (18 ADME and 12 non-ADME genes), their drug substrates and pharmacogenetic relevance were described in our previous study [12]. The metabolism of drugs is divided into three phases including modification (phase I), conjugation (phase II), and excretion (phase III). Thus, the ADME genes included: phase I, cytochrome P450 family (CYPs; *CYP2A6*,* CYP2B6*,* CYP2D6*,* CYP2C19*,* CYP2C8*,* CYP3A4*,* CYP3A5)*, phase II, N-acetyltransferase 2 (*NAT2*), UDP-glucuronosyltransferase family (*UGT1A1*) and phase III, ATP binding cassette subfamily B 1 (*ABCB1*), and solute carrier organic anion transporter family member 1B1 (*SLCO1B1*). The non-ADME genes included in the panel were: glucose-6-phosphate dehydrogenase (*G6PD*), nuclear factor Kappa B subunit 1 (*NF-κB1*), tumour necrosis factor (*TNF-α*), nuclear receptor subfamily 1, group I, member 2 (*NR1I2*), cut like homeobox 2 (*CUX2*) and ATP/GTP binding protein-like 4 (*AGBL4*).

### Genotyping of SNPs

Genomic DNA from blood was isolated using the QIAamp DNA Minikit (Qiagen, Hilden, Germany) according to the manufacturer’s instructions. The DNA quantity and quality were assessed with Nanodrop™ and Qubit 4 fluorometer (Thermo Fisher Scientific, Darmstadt, Germany). Genotyping of the selected SNPs was performed by allele discrimination TaqMan SNP genotyping assays [12] on a Thermo QuantStudio5™ real-time PCR system, 384-well (Thermo Fisher Scientific, Darmstadt, Germany) according to the manufacturer’s protocol. A 5 µL final reaction volume was used with a recommended DNA concentration of 0.2 ng/µL. The post-run data were loaded into the TaqMan Genotyper Software v1.5.0 (Thermo Fisher Scientific, Darmstadt, Germany) to retrieve the genotyping data automatically. The genotyping call rate was > 98%, with a 99% agreement with the randomly genotyped samples. The distribution of genotypes was subjected to possible deviations from Hardy-Weinberg equilibrium (HWE). In addition, the allele frequencies of 32 SNPs in the current study were compared to those reported in the non-African and African populations from the 1000 Genomes data.

### Statistical analysis

Socio-demographic data, clinical information and laboratory-generated data were coded, and all statistical analyses were performed with Statistical Package for Social Science (SPSS) software version 25.0 for Windows (IBM Corp., USA). Categorical variables (e.g. gender, BMI group, education, HIV stage, genotype and allele frequencies) were expressed as numbers and percentages. Chi-square (*χ*^*2*^) tests were used to compare socio-demographic variables between patient groups and to compare the observed and expected allele frequencies according to HWE. Odds Ratios (OR) were calculated to establish associations of investigated SNPs with DIH. Continuous variables (e.g. age, baseline Hb, WBC, AST, ALT, ALP, urea and creatinine) were reported as means ($\:\overline{x}$ ± SEM) and means were compared using one-way ANOVA. Multinomial logistic regression analysis was applied to evaluate associations between each independent variable and the risk of DIH. Statistical significance was set at *p* < 0.05.

## Results

### Characteristics of the study subjects

**Fig. 1:**
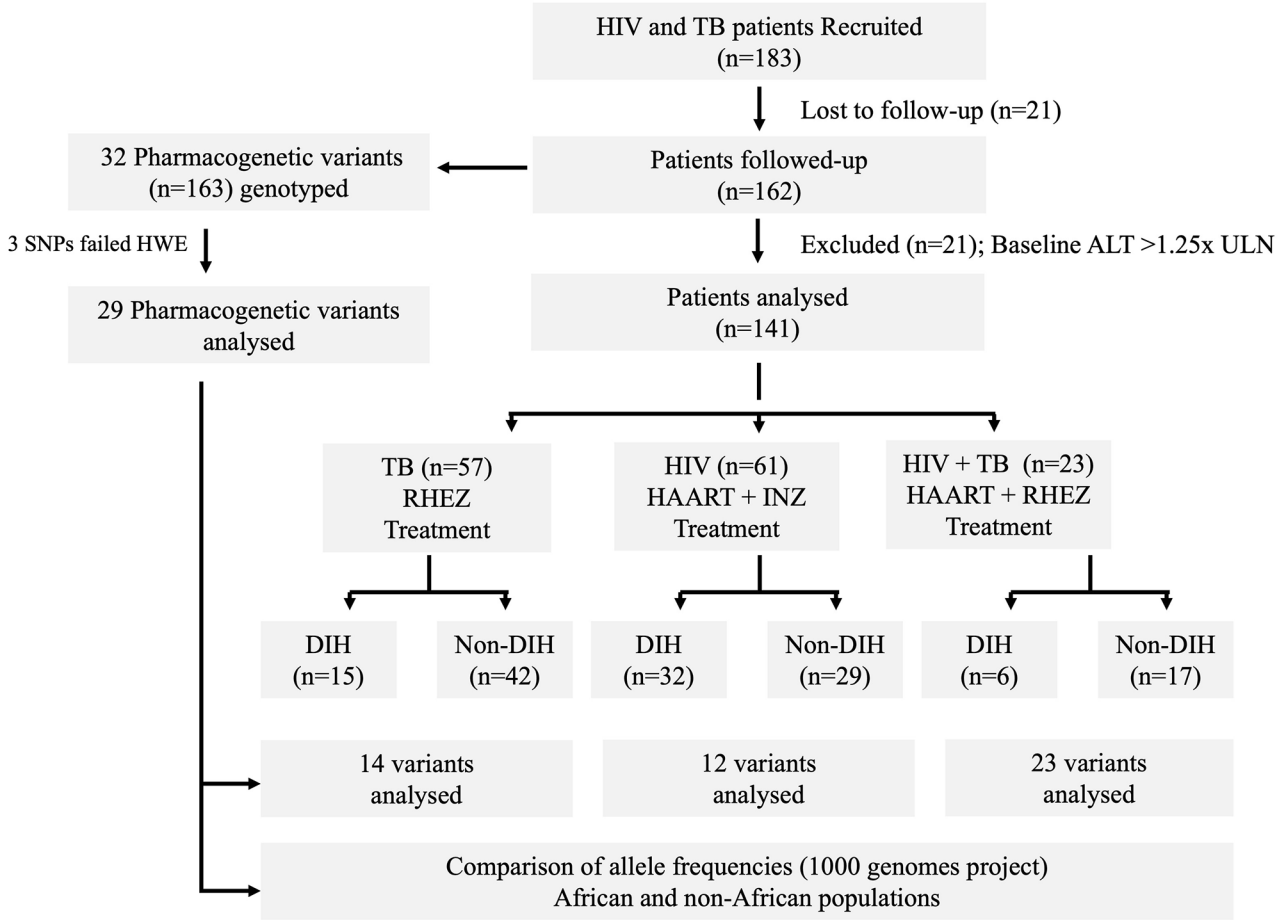
**Enrolled study population and follow-up of patients: **HWE (Hardy-Weinberg equilibrium); ALT (alanine aminotransferase); ULN (upper limit of normal); SNP (single nucleotide polymorphism); DIH (drug-induced hepatotoxicity); TB (tuberculosis); HIV (human immunodeficiency virus); HIV/TB (HIV and TB co-infection); ALT = >1.25x ULN; RHEZ regimen (rifampicin, isoniazid, ethambutol, pyrazinamide); HAART regimen (tenofovir disoproxil fumarate, lamivudine and efavirenz); INZ (isoniazid).

A total of 183 treatment-naïve patients with HIV, TB, or HIV/TB coinfection were initially recruited and treated with HAART, RHEZ or HAART plus RHEZ, respectively, and were followed up for 12 weeks. As 21 patients declined to participate, we included samples from 162 patients for genotyping and assessment of allele frequencies of selected pharmacogenetic variants to investigate the genetic predisposition for DIH. Out of the 162 patients, 21 study participants had elevated transaminases (ALT > 1.25 x ULN) and were subsequently excluded from further analyses (Fig. 1). Among the remaining 141 patients, 57 had TB, 61 were HIV-positive, and 23 were HIV/TB co-infected. We compared the socio-demographic, baseline clinical and laboratory parameters among TB, HIV and HIV/TB groups and observed that age, body mass index (BMI), baseline haemoglobin (Hb) and urea were significantly different among TB, HIV and HIV/TB groups (*P* < 0.05). Particularly, baseline Hb levels were lower while urea levels were higher in patients with HIV/TB compared to other groups. (Table 1).

**Table 1:** Demographic, clinical characteristics, and laboratory parameters of the population

Parameters		Patients (*n* = 141)			Total (%)	*p*-value
		**TB (%) ** *n* ** = 57**	**HIV (%)** *n* = 61	**HIV/TB (%)** *n* = 23		
Age (years)	≥ 40	20 (35)	31 (51)	12 (52)	63 (45)	0.167
	< 40	37 (65)	30 (49)	11 (48)	78 (55)	
	Age ($\:\overline{x}$ ± SEM)	36.4 ± 1.8	41.3 ± 1.3	42.3 ± 2.8	39.5 ± 1.1	**0.046**
Gender	Female	22 (39)	37 (61)	12 (52)	71 (50)	0.056
	Male	35 (61)	24 (39)	11 (48)	70 (50)	
BMI (Kg/m^2^)	Malnourished	7 (12)	2 (3)	6 (26)	15 (11)	**0.003**
	Overweight	14 (25)	30 (49)	5 (22)	49 (35)	
	Eutrophic	36 (63)	29 (48)	12 (52)	77 (55)	
	BMI ($\:\overline{x}$ ± SEM)	22.8 ± 0.5	25.5 ± 0.5	22.2 ± 1.2	23.9 ± 0.4	**0.001**
Education	Tertiary	19 (33)	10 (16)	1 (4)	30 (21)	**0.004**
	Secondary	15 (26)	33 (54)	13 (57)	61 (43)	
	Primary	23 (40)	18 (30)	9 (39)	50 (36)	
HIV stage	Stage 1	NA	22 (36)	6 (26)	28 (33)	0.332
	Stage 2	NA	27 (44)	9 (39)	36 (43)	
	Stage 3	NA	12 (20)	8 (35)	20 (24)	
Baseline Mean ($\:\overline{\varvec{x}}$ ±SEM)	HB (g/dL)	11.9 ± 0.3	11.3 ± 0.3	9.7 ± 0.7	11.3 ± 0.2	**0.001**
	WBC	5.9 ± 0.4	4.6 ± 0.5	5.8 ± 1.5	5.4 ± 0.4	0.199
	AST	39.8 ± 4.3	33.8 ± 3.1	33.7 ± 4.9	36.2 ± 2.3	0.447
	ALT	18.8 ± 1.4	19.9 ± 1.6	21.3 ± 3.2	19.7 ± 1.0	0.705
	ALP	179.5 ± 14.1	183.4 ± 14.7	228.2 ± 50.1	189.1 ± 11.8	0.342
	Urea	17.1 ± 1.2	23.4 ± 1.6	24.9 ± 2.6	21.1 ± 1.0	**0.004**
	Creatinine	1.0 ± 0.04	1.1 ± 0.06	1.1 ± 0.11	1.1 ± 0.03	0.116

TB-tuberculosis; HIV- human immunodeficiency virus; SEM- structural equation modelling; BMI- body mass index; NA- not applicable; HB- haemoglobin; WBC- white blood cells; AST- aspartate aminotransferase; ALT- alanine transaminase; ALP- alkaline phosphatase

### Drug-induced hepatotoxicity (DIH) during the 12-week follow-up

The overall cumulative incidence of DIH over the 12 weeks was 38% (Table 2). Among the patient groups, the cumulative incidence of DIH in the TB, HIV and HIV/TB patient groups was 26%, 52% and 26%, respectively (Table 2). The highest incidence of hepatotoxicity was observed in HIV patients treated with HAART plus isoniazid. Notably, we observed that 81% of patients with DIH had mild Grade 1 hepatotoxicity (Supplementary Table 1). Of 32 SNP variants found in the Cameroonian population, 29 SNPs were in HWE and therefore were used for further association analysis. Three other SNPs, namely rs2472677 (NR1I2), rs1050828, and rs1050829 (G6PD), failed the HWE assessment and therefore were excluded from further analysis.

**Table 2:** Drug induced hepatotoxicity with serum ALT levels expressed as multiples of the upper limit of the normal range in the study population

Patient	DIH *n* (%) ALT ≥ 1.25x ULN	Non-DIH *n* (%) ALT ≤ 1.25x ULN
TB, HIV, HIV + TB (*n* = 141)	53/141 (38)	88/141 (62)
TB (*n* = 57)	15/57 (26)	42/57 (74)
HIV (*n* = 61)	32/61 (52)	29/61 (48)
HIV + TB (*n* = 23)	6/23 (26)	17/23 (74)

DIH - Drug induced hepatotoxicity; TB-tuberculosis; HIV- human immunodeficiency virus; ALT- alanine transaminase; ULN- upper limit of the normal range

**Table 3:** Association of pharmacogenetic variants with drug-induced hepatotoxicity in tuberculosis patients treated with RHEZ.

ADME	Single Nucleotide Polymorphism (SNP)	Genotypes /Alleles	TB treatment		OR (95% CI)	*P* value
			**DIH (15 patients)** **n (%)**	**Non-DIH (42 patients)** **n (%)**		
Phase II metabolizing enzymes (ADME)	#rs1801280 NAT2*5 341T > C (I114T)	*TT*	3 (20)	22 (52)		**0.046**
		*CT*	10 (67)	18 (43)		
		*CC*	2 (13)	2 (5)		
		*T*	16 (53)	62 (74)	0.4 (0.17–0.96)	**0.038**
		*C*	14 (47)	22 (26)	1	
	#rs1799930 NAT2*6 590G > A (R197Q)	*GG*	12 (80)	21 (50)		0.110
		*AG*	3 (20)	15 (36)		
		*AA*	0 (0)	6 (14)		
		*G*	27 (90)	57 (68)	4.2 (1.1–15.2)	**0.017**
		*A*	3 (10)	27 (32)	1	
	#rs1799929 NAT2*11 481 C > T (L161L)	*CC*	5 (33)	25 (60)		0.143
		*TC*	8 (54)	15 (36)		
		*TT*	2 (13)	2 (5)		
		*C*	18 (60)	65 (77)	0.4 (0.2–1.1)	0.093
		*T*	12 (40)	19 (23)	1	
	#rs1208 NAT2*12 803 A > G (K268R)	*AA*	2 (13)	10 (24)		0.770
		*GA*	10 (67)	24 (57)		
		*GG*	3 (20)	8 (19)		
		*A*	14 (47)	44 (52)	0.8 (0.3–1.8)	0.672
		*G*	16 (53)	40 (48)	1	
	#rs1041983 NAT2*13 282 C > T (Y94Y)	*CC*	8 (53)	16 (38)		0.416
		*TC*	6 (40)	17 (41)		
		*TT*	1 (7)	9 (21)		
		*C*	22 (73)	49 (58)	1.9 (0.8–4.9)	0.189
		*T*	8 (27)	35 (42)	1	
	#rs1495741 *NAT2-18415371G > A* (Tag SNP)	*AA*	5 (38)	17 (42)		0.999
		*AG*	6 (46)	17 (42)		
		*GG*	2 (16)	7 (17)		
		*A*	10 (38)	31 (38)	1.0 (0.4–2.4)	1.000
		*G*	16 (62)	51 (62)	1	
Phase III transporters (ADME)	#rs2306283 SLCO1B1*1b 388 A > G (N130D)	*GG*	9 (60)	23 (56)		0.999
		*AG*	6 (40)	18 (44)		
		*AA*	0 (0)	0 (0)		
		*G*	24 (80)	64 (78)	1.1 (0.4–3.4)	1.000
		*A*	6 (20)	18 (22)	1	
	#rs4149032 *SLCO1B1c.85* *7793 C > T* (Intron)	*TT*	6 (43)	16 (39)		0.536
		*CT*	8 (57)	20 (49)		
		*CC*	0 (0)	5 (12)		
		*T*	20 (71)	52 (63)	1.4 (0.6–3.7)	0.497
		*C*	8 (29)	30 (37)	1	
	#rs1045642 ABCB1*6 3435 C > T (I1145I)	*CC*	13 (87)	32 (76)		0.485
		*TC*	2 (13)	10 (24)		
		*TT*	0 (0)	0 (0)		
		*C*	28 (93)	74 (88)	1.9 (0.4–9.2)	0.724
		*T*	2 (7)	10 (12)	1	
Non-ADME	#rs4647992 - *NFκB1_c.159* *+ 305 C > T* (Intron)	*CC*	13 (87)	35 (83)		1.000
		*TC*	2 (13)	7 (17)		
		*TT*	0 (0)	0 (0)		
		*C*	28 (93)	77 (92)	1.3 (0.2–6.4)	1.000
		*T*	2 (7)	7 (8)	1	
	#rs1800629 *TNF-α* *-308G > A* (Regulatory)	*GG*	12 (80)	36 (86)		0.685
		*AG*	3 (20)	6 (14)		
		*AA*	0 (0)	0 (0)		
		*G*	27 (90)	78 (93)	0.7 (0.2–3.0)	0.696
		*A*	3 (10)	6 (7)	1	
	#rs7958375 *CUX2_c.64* *11987G > A* (Intron)	*GG*	11 (73)	25 (59)		0.655
		*GA*	4 (27)	16 (38)		
		*AA*	0 (0)	1 (2)		
		*G*	26 (87)	66 (79)	1.8 (0.5–5.7)	0.425
		*A*	4 (13)	18 (21)	1	
	#rs319952 *AGBL4_c.206* *+ 5387T > C* (Intron)	*TT*	2 (13)	0 (0)		0.470
		*CT*	5 (33)	18 (43)		
		*CC*	8 (53)	24 (57)		
		*T*	21 (70)	66 (79)	0.6 (0.2–1.6)	0.453
		*C*	9 (30)	18 (21)	1	
	#rs320003 *AGBL4_c.91* *+ 2046 C > T* (Intron)	*CC*	6 (40)	26 (62)		0.109
		*CT*	8 (53)	16 (38)		
		*TT*	1 (7)	0 (0)		
		*C*	20 (67)	68 (81)	0.5 (0.2–1.2)	0.130
		*T*	10 (33)	16 (19)	1	

ADME: Absorption, distribution, metabolism, and excretion. The metabolism of drugs is divided into three phases including modification (phase I), conjugation (phase II), and excretion (phase III). n (%): the numbers of observed genotypes/alleles and corresponding percentage

**Table 4:** Examined 32 SNPs and the respective allele frequencies (%) in the current study compared to those reported in the non-african and African populations from the 1000 genomes data

				Non-African population				African population							
	**Gene**	#**SNP ID**	**Risk allele**	**AMR**	**EAS**	**EUR**	**SAS**	**ACB**	**ASW**	**ESN**	**GWD**	**LWK**	**MSL**	**YRI**	**CMR** (study)
Phase I metabolizing enzymes (ADME)	***CYP2A6***	rs28399433	*G*	10	**24**	7	**15**	6	11	10	6	9	7	10	7
	***CYP2B6***	rs28399499	*C*	1	0	0	0	6	10	7	10	6	8	12	11
		rs3745274	*T*	**37**	**22**	**24**	38	38	**35**	41	**35**	**36**	**35**	40	42
	***CYP2D6***	rs1065852	*T*	**15**	**57**	**20**	**17**	**15**	**16**	9	**12**	4	**17**	**11**	6
	***CYP2C19***	rs12768009	*A*	10	**37**	15	**37**	16	14	22	14	**23**	19	17	18
		rs4244285	*A*	**11**	**31**	15	**36**	15	**14**	21	**13**	21	18	17	19
	***CYP2C8***	rs11572103	*A*	**1**	**0**	**0**	**1**	**21**	**15**	20	**24**	14	17	20	16
	***CYP3A4***	rs2740574	*G*	**10**	**0**	**3**	**4**	**66**	**67**	77	79	83	83	**76**	81
	***CYP3A5***	rs776746	*G*	**20**	**29**	**6**	**33**	**75**	**69**	**89**	**77**	**88**	**88**	83	83
		rs10264272	*A*	**2**	**0**	**0**	**0**	12	**5**	14	16	**24**	16	17	13
		rs41303343	*AA*	**0**	**0**	**0**	**0**	**11**	12	**9**	14	12	14	12	16
Phase II metabolizing enzymes (ADME)	***NAT2***	rs1801280	*C*	**36**	**4**	**45**	**35**	28	31	27	**34**	**36**	24	24	28
		rs1799930	*A*	**17**	26	28	**36**	26	29	26	19	27	22	**20**	26
		rs1799929	*T*	**34**	**4**	**44**	**32**	25	26	**19**	28	**33**	**19**	**17**	25
		rs1208	*G*	**37**	**4**	44	**36**	**38**	**36**	39	44	46	**35**	**37**	43
		rs1041983	*T*	**29**	44	**31**	43	**50**	45	**53**	39	43	**50**	**50**	42
		rs1495741	*A*	65	**48**	**76**	**78**	59	**67**	**56**	**57**	**66**	**54**	**51**	61
	***UGT1A1***	rs3064744	*(TA)8*	36	**13**	**29**	40	43	42	40	38	**46**	41	**46**	41
		rs10929303	*T*	**22**	**13**	**23**	**17**	46	39	**50**	47	**32**	**33**	45	43
	***UGT2B7***	rs7439366	*T*	**32**	28	**49**	**40**	28	25	24	**19**	25	**18**	**21**	26
Phase III transporters (ADME)	***SLCO1B1***	rs2306283	*A*	**53**	**24**	**60**	**45**	**21**	**25**	**12**	19	16	19	19	16
		rs4149032	*C*	**61**	**40**	**66**	**49**	30	**36**	**19**	23	28	27	28	27
	***ABCB1***	rs1045642	*T*	**43**	**40**	**52**	**58**	**15**	**19**	12	**19**	14	**15**	13	10
		rs3842	*G*	**16**	**30**	**14**	18	**4**	17	17	**12**	**25**	**16**	**15**	21
Non-ADME	***G6PD*** ^***$***^	rs1050828	*A*	1	0	0	0	13	17	16	4	18	7	21	6
		rs1050829	*G*	3	0	0	0	33	29	35	36	34	28	38	22
	***NF-κB1***	rs4647992	*T*	**3**	**4**	5	**4**	10	9	10	8	**24**	8	10	9
	***TNF*** *-α*	rs1800629	*A*	7	6	13	**5**	14	7	13	14	9	**16**	10	10
	***NR1I2*** ^***$***^	rs2472677	*T*	48	62	66	57	42	35	36	38	40	28	36	37
	***CUX2***	rs7958375	*G*	**98**	**100**	**100**	**99**	83	86	85	84	79	**76**	81	81
	***AGBL4***	rs319952	*T*	**51**	67	**40**	**46**	70	**63**	71	80	**67**	**78**	74	71
		rs320003	*C*	**51**	**67**	**40**	**46**	**69**	**64**	75	**80**	74	**80**	74	74

ADME-absorption, distribution, metabolism and excretion; The metabolism of drugs is divided into three phases including modification (phase I), conjugation (phase II), and excretion (phase III). Bold- allele frequency ≥ 5% compared to the Cameroonian population. Allele frequencies were retrieved from www.ensembl.org. ^$^SNPs that are not in HWE in this study. African (AFR), Yoruba in Ibadan Nigeria (YRI), Luhya in Webuye Kenya (LWK), Gambians in the West Coast Region (GWD), Mende in Sierra Leone (MSL), Esan in Nigeria (ESN), Americans of African Ancestry in SW USA (ASW), African Caribbeans in Barbados (ACB), Mixed American (AMR), East Asian (EAS), European (EUR), South Asian (SAS) and Cameroonian (CMR).

### Association of pharmacogenetic variants with drug-induced hepatotoxicity

The genetic predisposition for DIH results investigated for each patient group are shown in Table 3 and in supplementary Tables 2 and 3. To decipher the genetic predisposition for DIH in TB patients taking TB drugs, we performed an association analysis of 14 pharmacogenetic variants in genes *NAT2*,* ABCB1*,* SLC01B1*,* NF-κB1*,* TNF-α*,* CUX2* and *AGBL4*, and the results show that the investigated variants are reported to be associated with DIH and ADRs (Table 3). Particularly, genotype distritution of slow acetylation NAT2*5 vatiant was significantly distributed between patients with and without DIH among those treated for TB (*p* = 0.046). The NAT2*5 allele *T* was associated with decreased risk of DIH (OR = 0.4; 95% CI = 0.17–0.96; *p* = 0.038), while the NAT2*6 allele *G* were associated with increased risk of DIH among those treated for TB (OR = 4.2; 95% CI = 1.1–5.2; *p* = 0.017) (Table 3).

The association analysis results showed that none of the 12 pharmacogenetic variants of genes *CYP2A6*,* CYP2B6*,* CYP3A4*,* CYP3A5*,* UGT2B7* and *ABCB1*, which have previously shown to be associated with HIV-treatment induced DIH or ADRs [13], was associated with DIH in HIV patients (Supplementary Table 2). Subsequently, among 23 pharmacogenetic variants in genes *CYP2A6*,* CYP2B6*,* CYP3A4*,* CYP3A5*,* UGT2B7*,* NAT2*,* ABCB1*,* SLC01B1*,* NF-κB1*,* TNF-α*,* CUX2* and *AGBL4*, we found the non-ADME variant rs4647992 (*NF-kB1*) minor allele *T* associated with DIH in HIV/TB patients (*p* = 0.032) (Supplementary Table 3).

### Allele frequencies in African and non-African populations

The analysis is based on a ≥ 5% deviation in allele frequencies within the Cameroonian (CMR) population. We conducted paired comparisons of 29 SNPs in the CMR population with other African populations. SNPs deviating ≥ 5% in allele frequencies are shown in bold (Table 4). Polymorphisms in African populations that differed from CMR population by ≥ 5% include 11 polymorphisms in Caribbean people of African descent (ACB), 14 polymorphisms in Americans in the South-West United States (ASW), eight polymorphisms in the Esan in Nigeria (ESN), 11 polymorphisms in Gambians in the West Coast Region (GWD), 13 polymorphisms in the Luhya in Webuye, Kenya (LWK), 15 polymorphisms in the Mende in Sierra Leone (MSL) and ten polymorphisms in the Yoruba in Ibadan, Nigeria (YRI) population.

A pair-wise comparison of 29 SNPs in the CMR population with the non-African populations was performed. SNPs that deviated ≥ 5% in allele frequencies are shown in bold and underlined (Table 4). The number of polymorphisms in the African populations that differed from the CMR population by ≥ 5% included 25 polymorphisms in the SAS, 24 polymorphisms in the mixed American (AMR), 24 polymorphisms in the East Asian (EAS), and 22 polymorphisms in the European (EUR) populations. Among the 29 SNPs investigated in the CMR population, we observed that eight to 15 SNPs differed in ≥ 5% of allele frequencies among African populations, while 22 to 25 SNPs differed in ≥ 5% of the allele frequencies among non-African populations, respectively (Table 4).

## Discussion

We explored pharmacogenetic variants in ADME genes that can predict DIH, to advise the dosing regimens to be used in each case. Specifically in this prospective study, we genotyped 29 clinically relevant pharmacogenetic variants that have been reported to be associated with DIH/ADRs for the drugs used to treat HIV and TB. This study marks the first comprehensive study in Cameroon to identify allelic frequencies of potentially clinically relevant pharmacogenetic variants. We found that the overall incidence of DIH was 38% mainly consisting of Grade 1, which is considered mild and aligns with findings from other studies [14], not requiring any intervention. Approximately 26% of the TB patients treated with TB drugs developed hepatotoxicity. DIH incidences in other TB studies have shown a wide range from 2 to 28% [15]. These variations are attributed to individual, ethnic and genetic factors, as well as the respective definitions applied to classify hepatotoxicity. In other studies, DIH associated with HAART treatment ranged from 1 to 20% [16]. Furthermore, about 26% of HIV/TB co-infected patients in our study developed DIH when taking TB and efavirenz-based HAART drug regimens. Reports have shown DIH as the most common adverse event reported in the treatment of TB in HIV with rates ranging from 11 to 30% [14, 17].

Slow *NAT2* acetylation variants were found to have a significant association with DIH caused by TB drugs. NATs are responsible for the acetylation of arylamine drugs, and several *NAT2* variants are associated with a slow acetylation phenotype, while others lead to a fast acetylation phenotype [18]. Isoniazid, in particular, is metabolised by the NAT2 enzyme [19] and the slow acetylation variants are known to be associated with TB DIH [20, 21]. A recent study in Ethiopian TB patients has shown that *NAT2* genotype and acetylation status were correlated with plasma isoniazid concentration suggesting that *NAT2* acetylation status are promising predictors of isoniazid plasma concentrations [22]. The *NAT2* variants can be classified based on their acetylation activity (NAT2*4 > NAT2*7 > NAT2*6 > NAT2*5) [18]. NAT2*5, NAT2*6, and NAT2*7 are the main alleles associated with reduced enzyme activity, exhibiting the slow acetylator phenotype. In this study, we identified NAT2*5 and NAT2*6 variants associated with DIH in TB treatment, indicating that the Cameroonian population has a genetic predisposition to DIH. Previous meta-analyses have also shown a strong association of these variants with DIH during TB treatment [23, 24]. Due to their vital clinical significance, these variants could be employed to predict DIH in the Cameroonian population. Our study is the first to demonstrate the association of *NAT*2 gene variants with DIH in this population; and because of their clinical relevance, *NAT* variants are recommended for pharmacogenomic labelling (www.PharmGKB.org). Specifically, for individuals with slow acetylator genotype *NAT2*, screening for these polymorphisms could offer significant clinical benefits by identifying patients at a high risk of developing DIH during TB therapy. This information can aid in minimizing the risk through the application of alternative drugs [24].

In the association analysis, we observed a statistically significant association between the non-ADME variant rs4647992 (*NF-kB*) and DIH in HIV/TB co-infected patients undergoing HIV plus TB treatment. NF-κB1 is a transcription factor known for its essential role in inflammatory signalling pathways and its contribution to liver homeostasis and healing [25]. NF-κB also regulates genes involved in drug-induced liver damage such as *UGT1A1* and *NR1I2*. Although the association of rs4647992 with DIH has been reported in only a few studies [26], further studies are needed to validate the clinical significance.

Cytochromes P450 (CYPs) represent the most important class of ADME enzymes involved in the metabolism of 70–80% of drugs. Several studies in the Cameroonian population have investigated some of these variants, including CYP2B6*6, CYP3A4*1B, CYP3A5*3, and CYP3A5*6 [27–29]. Among these, the CYP2B6*6 gene has been extensively studied with regard to its established clinical significance in efavirenz- and nevirapine-induced ADRs/DIH in African populations [30]. The allele frequency of CYP2B6*6 in Yaoundé, in Bantu-speaking and Djang populations of Cameroon, is reported to be 59%, 37%, and 44%, respectively [27–29]. Our data indicate that the frequency of this variant is 42%, which is close to the frequency in Bantu-speaking and Djang populations. However, this significantly differs from the Yaoundé population, where the frequency is 59%. This observation underscores the existence of genetic differences between different ethnic groups in the region.

The variant CYP3A4*1B has been associated with efavirenz, atazanavir, lumefantrine, and indinavir; drug-induced ADRs [31]. Similarly, the CYP3A5*3 and CYP3A5*6 variants are associated with lumefantrine, atazanavir, ritonavir, and nevirapine-induced ADRs [31]. The allele frequencies of CYP3A4*1B, CYP3A5*3 and CYP3A5*6 in the Bantu-speaking population of Cameroon are reported to be 78%, 17%, and 16%, respectively [27]. In this study, the allele frequencies observed in the Fako Division of Cameroon were 83%, 13%, and 16%, respectively. The allele frequencies of CYP3A4*1B and CYP3A5*3 variants in both the Bantu-speaking population and the Fako Division vary between 4 and 5%, indicating that not much difference exists between these populations. Overall, information on the variant´s allele frequencies shall be valuable in establishing dosage guidelines and understanding interindividual variation when administering medications.

The study remains with certain limitations. Firstly, the relatively small sample size does not provide sufficient power to replicate previously associated variants. Secondly, we did not include the variants HLA-B*57:01:01, rs2032582 (*ABCB1*), rs1799931 (*NAT2*), rs717620 (*ABCC2*) and rs10946737 (*RIPOR2*) (www.PharmGKB.org), with allele frequencies below 10%, despite their strong association with HIV- or TB treatment-induced DIH/ADRs, due to technical limitations in constructing the SNP genotyping panel, and selected variants had allele frequencies > 10% known to occur in African populations. Thirdly, we could not analyse the association between the genetic variants with drug metabolism due to the lack of TB and HIV drug concentration data.

## Conclusions

Taken together, most DIH events in the study population were mild, with the highest toxicity observed among HIV patients undergoing HAART therapy and isoniazid prophylaxis. The NAT2*5 and NAT2*6 variants were found to be associated with DIH caused by TB drugs in the Cameroonian population, and these variants can serve as valuable biomarkers. Translation of pharmacogenetic and pharmacogenomic research into clinical practice has been rather slow, but there is an increasing approval of pharmacogenomic drug labels. This first study will significantly contribute to a better understanding of the role of pharmacogenetic variants in drug responses. It will enable the prediction of drug efficacy, toxicity, and ADRs, ultimately supporting individual and population-based decisions regarding drug treatment.

### Electronic supplementary material

Below is the link to the electronic supplementary material.

Supplementary Material 1

## Data Availability

All data generated or analysed during this study are included in this published article and its supplementary information files.

## Abbreviations

HIV: Human immunodeficiency virus
AIDS: acquired immunodeficiency syndrome
TB: tuberculosis
SSA: sub-Saharan Africa
ADR: adverse drug reactions. DIH: drug-induced hepatotoxicity
ADME: absorption, distribution, metabolism and excretion
ALT: alanine aminotransaminase
AST: aspartate aminotransaminase
γ-GT: gamma-glutamyl transferase
ALP: alkaline phosphatase
