# Supplementary material for: Drug-induced hepatotoxicity and association with slow acetylation variants NAT2*5 and NAT2*6 in Cameroonian patients with tuberculosis and HIV co-infection
Source: BMC Infect Dis. 2024 Jul 31;24:759. doi: 10.1186/s12879-024-09638-w (PMC11293078; doi:10.1186/s12879-024-09638-w)
Supplement: Supplementary file 1 — Supplementary Material 1 [file 12879_2024_9638_MOESM1_ESM.docx]

**Supplementary Data**

Supplementary Table 1: Severity of liver injury in patients during 12 weeks follow-up

| **Level of severity** | **Duration of follow-up** | | | | |
| --- | --- | --- | --- | --- | --- |
|  | **Baseline**  **n (%)** | **Week 1**  **n (%)** | **Week 4**  **n (%)** | **Week 8**  **n (%)** | **Week 12**  **n (%)** |
| Grade 0 (No injury) | 141 (87) | 126 (89) | 92 (76) | 73 (76) | 34 (69) |
| Grade 1 (Mild injury) | 21 (13) | 13 (9) | 26 (22) | 16 (17) | 12 (25) |
| Grade 2 (Moderate injury) | - | 1 (1) | 3 (3) | 6 (6) | 3 (6) |
| Grade 3 (Severe injury) | - | 1 (1) | - | 1 (1) | - |
| Incidence (%) | 21 (13) | 15 (11) | 29 (24) | 23 (24) | 15 (31) |
| Total | 162 | 141 | 138 | 112 | 57 |

**Supplementary Table 2: Association of SNP variants for HIV treatment drug-induced hepatotoxicity**

|  |  | **HIV treatment** | |  | **Fisher exact** |
| --- | --- | --- | --- | --- | --- |
| **SNP** | **Genotype/Allele** | **DIH (32 patients)**  **n (%)** | **Non-DIH (29 patients)**  **n (%)** | **OR (95% CI)** | ***p*-value** |
| **Phase I (ADME)** | | | | | |
| rs28399433 - CYP2A6*9_-48T>G (Promoter) | | | | | |
|  | TT | 28 (87) | 24 (83) | N/A | 0.724 |
|  | GT | 4 (13) | 5 (17) |  |  |
|  | GG | 0 (0) | 0 (0) |  |  |
|  | T | 60 (94) | 53 (91) | 1.4 (0.4 - 5.5) | 0.734 |
|  | G | 4 (6) | 5 (9) | 1 |  |
| rs28399499 - CYP2B6*18_c.983T>C(I328T) | | | | | |
|  | TT | 24 (75) | 23 (79) | N/A | 0.541 |
|  | CT | 8 (25) | 5 (17) |  |  |
|  | CC | 0 (0) | 1 (4) |  |  |
|  | T | 56 (87) | 51 (88) | 1 (0.3 -2.8) | 1.000 |
|  | C | 8 (13) | 7 (12) | 1 |  |
| rs3745274 - CYP2B6*6_c.516G>T (Q172H) | | | | | |
|  | GG | 12 (37) | 10 (34) | N/A | 0.834 |
|  | TG | 14 (44) | 15 (52) |  |  |
|  | TT | 6 (19) | 4 (14) |  |  |
|  | G | 38 (59) | 35 (60) | 1 (0.5 - 2.0) | 1.000 |
|  | T | 26 (51) | 23 (40) | 1 |  |
| rs2740574 - CYP3A4*1B_-392A>G (Intergenic) | | | | | |
|  | GG | 18 (56) | 12 (41) | N/A | 0.424 |
|  | AG | 12 (38) | 16 (55) |  |  |
|  | AA | 2 (6) | 1 (4) |  |  |
|  | G | 48 (75) | 40 (69) | 1.3 (0.6 - 2.9) | 0.545 |
|  | A | 16 (25) | 18 (31) | 1 |  |
| rs776746 - CYP3A5*3_6986A>G (Splice defect) | | | | | |
|  | GG | 20 (62) | 20 (69) | N/A | 0.787 |
|  | GA | 12 (38) | 9 (31) |  |  |
|  | AA | 0 (0) | 0 (0) |  |  |
|  | G | 52 (81) | 49 (84) | 0.8 (0.3 - 1.8) | 0.810 |
|  | A | 12 (19) | 9 (16) | 1 |  |
| rs10264272 - CYP3A5*6_14690G>A (K208K) | | | | | |
|  | GG | 23 (72) | 24 (83) | N/A | 0.371 |
|  | AG | 9 (28) | 5 (17) |  |  |
|  | AA | 0 (0) | 0 (0) |  |  |
|  | G | 55 (86) | 53 (91) | 0.6 (0.2 - 1.8) | 0.404 |
|  | A | 9 (14) | 5 (9) | 1 |  |
| rs41303343 - CYP3A5*7_27132insA (-346S) | | | | | |
|  | A\|A | 24 (75) | 17 (59) | N/A | 0.205 |
|  | AA\|A | 8 (25) | 10 (34) |  |  |
|  | AA\|AA | 0 (0) | 2 (7) |  |  |
|  | A | 56 (87) | 44 (76) | 2.2 (0.9 to 5.8) | 0.105 |
|  | AA | 8 (13) | 14 (24) | 1 |  |
| **Phase II (ADME)** | | | | | |
| rs7439366 - UGT2B7*2_2100C>T (Y268H) | | | | | |
|  | CC | 20 (63) | 13 (45) | N/A | 0.351 |
|  | TC | 11 (34) | 15 (52) |  |  |
|  | TT | 1 (3) | 1 (3) |  |  |
|  | C | 51 (80) | 41 (71) | 1.6 (0.7 - 3.7) | 0.295 |
|  | T | 13 (20) | 17 (29) | 1 |  |
| **Phase III (ADME)** | | | | | |
| rs2306283 - SLCO1B1*1b_388A>G (N130D) | | | | | |
|  | GG | 24 (76) | 22 (76) | N/A | 1.000 |
|  | AG | 7 (21) | 7 (24) |  |  |
|  | AA | 1 (3) | 0 (0) |  |  |
|  | G | 55 (86) | 51 (88) | 0.8 (0.3 - 2.4) | 0.793 |
|  | A | 9 (14) | 7 (12) | 1 |  |
| rs4149032 - SLCO1B1_c.85-7793C>T (Intron) | | | | | |
|  | TT | 19 (59) | 17 (59) | N/A | 0.528 |
|  | CT | 11 (34) | 12 (41) |  |  |
|  | CC | 2 (7) | 0 (0) |  |  |
|  | T | 49 (77) | 46 (79) | 0.8 (0.4 - 2.0) | 0.828 |
|  | C | 15 (23) | 12 (21) | 1 |  |
| rs1045642 - ABCB1*6_3435C>T (I1145I) | | | | | |
|  | CC | 27 (84) | 25 (86) | N/A | 0.999 |
|  | TC | 4 (13) | 4 (14) |  |  |
|  | TT | 1 (3) | 0 (0) |  |  |
|  | C | 58 (91) | 54 (93) | 0.7 (0.2 - 2.7) | 0.746 |
|  | T | 6 (9) | 4 (7) | 1 |  |
| rs3842 - ABCB1_c.193A>G (3'UTR) | | | | | |
|  | AA | 23 (72) | 19 (65) | N/A | 0.909 |
|  | GA | 7 (22) | 8 (28) |  |  |
|  | GG | 2 (6) | 2 (7) |  |  |
|  | A | 53 (83) | 46 (79) | 1.3 (0.5 - 3.1) | 0.649 |
|  | G | 11 (17) | 12 (21) | 1 |  |

N/A: Not applicable

ADME: Absorption, distribution, metabolism and excretion. The metabolism of drugs is divided into three phases including modification (phase I), conjugation (phase II), and excretion (phase III). n: the numbers of observed genotypes/alleles and corresponding percentage.

**Supplementary Table 3: Association of SNP variants for HIV/TB treatment drug-induced hepatotoxicity**

|  |  | **HIV/TB treatment** | |  | **Fisher exact** |
| --- | --- | --- | --- | --- | --- |
| **SNP** | **Genotype/Allele** | **DIH (6 patients)**  **n (%)** | **Non-DIH (17 patients)**  **n (%)** | **OR (95% CI)** | ***p*-value** |
| **Phase I (ADME)** | | | | | |
| rs28399433 - CYP2A6*9_-48T>G (Promoter) | | | | | |
|  | TT | 6 (100) | 14 (82) | N/A | 0.539 |
|  | GT | 0 (0) | 3 (18) |  |  |
|  | GG | 0 (0) | 0 (0) |  |  |
|  | T | 12 (100) | 31 (91) | 2.7 (0.1-57) | 0.556 |
|  | G | 0 (0) | 3 (9) | 1 |  |
| rs28399499 - CYP2B6*18_c.983T>C (I328T) | | | | | |
|  | TT | 6 (100) | 14 (82) | N/A | 0.539 |
|  | CT | 0 (0) | 3 (18) |  |  |
|  | CC | 0 (0) | 0 (0) |  |  |
|  | T | 12 (100) | 31 (91) | 2.7 (0.1-57) | 0.556 |
|  | C | 0 (0) | 3 (9) | 1 |  |
| rs3745274 - CYP2B6*6_c.516G>T (Q172H) | | | | | |
|  | GG | 2 (33) | 4 (24) | N/A | 1.000 |
|  | TG | 3 (50) | 10 (59) |  |  |
|  | TT | 1 (17) | 3 (18) |  |  |
|  | G | 7 (58) | 18 (53) | 1.2 (0.3 - 4.7) | 1.000 |
|  | T | 5 (42) | 16 (47) | 1 |  |
| rs2740574 - CYP3A4*1B_-392A>G (Intergenic) | | | | | |
|  | GG | 5 (83) | 11 (65) | N/A | 0.621 |
|  | AG | 1 (17) | 6 (35) |  |  |
|  | AA | 0 (0) | 0 (0) |  |  |
|  | G | 11 (92) | 28 (82) | 2.3 (0.3 - 21.8) | 0.656 |
|  | A | 1 (8) | 6 (18) | 1 |  |
| rs776746 - CYP3A5*3_6986A>G (Splice defect) | | | | | |
|  | GG | 6 (100) | 12 (71) | N/A | 0.660 |
|  | GA | 0 (0) | 4 (24) |  |  |
|  | AA | 0 (0) | 1 (6) |  |  |
|  | G | 12 (100) | 28 (82) | 5.7 (0.3 - 109) | 0.316 |
|  | A | 0 (0) | 6 (18) | 1 |  |
| rs10264272 - CYP3A5*6_14690G>A (K208K) | | | | | |
|  | GG | 3 (50) | 15 (88) | N/A | 0.088 |
|  | AG | 3 (50) | 2 (12) |  |  |
|  | AA | 0 (0) | 0 (0) |  |  |
|  | G | 9 (75) | 32 (94) | 0.2 (0.2 - 1.2) | 0.102 |
|  | A | 3 (25) | 2 (6) | 1 |  |
| rs41303343 - CYP3A5*7_27132insA (-346S) | | | | | |
|  | A\|A | 3 (50) | 12 (71) | N/A | 0.621 |
|  | AA\|A | 3 (50) | 5 (29) |  |  |
|  | AA\|AA | 0 (0) | 0 (0) |  |  |
|  | A | 9 (75) | 29 (85) | 0.5 (0.1 - 2.6) | 0.412 |
|  | AA | 3 (25) | 5 (15) | 1 |  |
| **Phase II (ADME)** | | | | | |
| rs1801280 - NAT2*5_341T>C (I114T) | | | | | |
|  | TT | 3 (50) | 11 (65) | N/A | 0.391 |
|  | CT | 3 (50) | 3 (18) |  |  |
|  | CC | 0 (0) | 3 (18) |  |  |
|  | T | 9 (75) | 25 (76) | 1 (0.2 - 5) | 1.000 |
|  | C | 3 (25) | 9 (26) | 1 |  |
| rs1799930 - NAT2*6_590G>A (R197Q) | | | | | |
|  | GG | 3 (50) | 12 (71) | N/A | 0.507 |
|  | AG | 3 (50) | 4 (24) |  |  |
|  | AA | 0 (0) | 1 (6) |  |  |
|  | G | 9 (75) | 28 (82) | 0.6 (0.1 - 3.1) | 0.677 |
|  | A | 3 (25) | 6 (18) | 1 |  |
| rs1799929 - NAT2*11_481C>T (L161L) | | | | | |
|  | CC | 3 (50) | 11 (65) | N/A | 0.362 |
|  | TC | 3 (50) | 4 (24) |  |  |
|  | TT | 0 (0) | 2 (12) |  |  |
|  | C | 9 (75) | 26 (76) | 0.9 (0.2 - 4.2) | 1.000 |
|  | T | 3 (25) | 8 (24) | 1 |  |
| rs1208 - NAT2*12_ 803A>G (K268R) | | | | | |
|  | AA | 3 (50) | 8 (47) | N/A | 0.999 |
|  | GA | 2 (33) | 4 (24) |  |  |
|  | GG | 1 (17) | 5 (29) |  |  |
|  | A | 8 (67) | 20 (59) | 0.7 (0.2 - 2.8) | 0.739 |
|  | G | 4 (33) | 14 (41) | 1 |  |
| rs1041983 - NAT2*13_282C>T (Y94Y) | | | | | |
|  | CC | 1 (17) | 8 (47) | N/A | 0.572 |
|  | TC | 3 (50) | 6 (35) |  |  |
|  | TT | 2 (33) | 3 (18) |  |  |
|  | C | 5 (42) | 22 (65) | 0.4 (0.1 – 1.4) | 0.190 |
|  | T | 7 (58) | 12 (35) | 1 |  |
| rs1495741 - NAT2_18415371G>A (Tag SNP) | | | | | |
|  | AA | 1 (17) | 5 (29) | N/A | 0.583 |
|  | AG | 4 (67) | 7 (41) |  |  |
|  | GG | 1 (17) | 5 (29) |  |  |
|  | A | 6 (50) | 17 (50) | 1 (0.3 - 3.7) | 1.000 |
|  | G | 6 (50) | 17 (50) | 1 |  |
| rs7439366 - UGT2B7*2_2100C>T (Y268H) | | | | | |
|  | CC | 4 (67) | 5 (29) | N/A | 0.285 |
|  | CT | 2 (33) | 10 (59) |  |  |
|  | TT | 0 (0) | 2 (12) |  |  |
|  | C | 10 (83) | 20 (59) | 3.5 (0.7 - 18.5) | 0.169 |
|  | T | 2 (17) | 14 (41) | 1 |  |
| **Phase III (ADME)** | | | | | |
| rs2306283 - SLCO1B1*1b_388A>G(N130D) | | | | | |
|  | GG | 6 (100) | 11(65) | N/A | 0.457 |
|  | AG | 0 (0) | 5 (29) |  |  |
|  | AA | 0 (0) | 1 (6) |  |  |
|  | G | 12 (100) | 27 (79) | 6.8 (0.4 - 128) | 0.164 |
|  | A | 0 (0) | 7 (21) | 1 |  |
| rs4149032 - SLCO1B1_c.85-7793C>T (Intron) | | | | | |
|  | T/T | 5 (83) | 10 (59) | N/A | 0.716 |
|  | C/T | 1 (17) | 6 (35) |  |  |
|  | C/C | 0 (0) | 1 (6) |  |  |
|  | T | 11 (92) | 26 (76) | 3.4 (0.4 - 30) | 0.409 |
|  | C | 1 (8) | 8 (24) | 1 |  |
| rs1045642 - ABCB1*6_3435C>T (I1145I) | | | | | |
|  | CC | 5 (83) | 13 (76) | N/A | 0.999 |
|  | TC | 1 (17) | 4 (24) |  |  |
|  | TT | 0 (0) | 0 (0) |  |  |
|  | C | 11 (92) | 30 (88) | 1.5 (0.1 - 15) | 1.000 |
|  | T | 1 (8) | 4 (12) | 1 |  |
| rs3842 - ABCB1_c193A>G (3'UTR) | | | | | |
|  | AA | 4 (67) | 8 (47) | N/A | 0.816 |
|  | GA | 2 (33) | 7 (41) |  |  |
|  | GG | 0 (0) | 2 (12) |  |  |
|  | A | 10 (83) | 23 (68) | 2.4 (0.4 - 13) | 0.461 |
|  | G | 2 (17) | 11 (32) | 1 |  |
| **Non-ADME** | | | | | |
| rs4647992 - NFκB1_c.159+305C>T (Intron) | | | | | |
|  | CC | 4 (67) | 15 (88) | N/A | 0.270 |
|  | TC | 2 (33) | 2 (12) |  |  |
|  | TT | 0 (0) | 0 (0) |  |  |
|  | C | 8 (67) | 32 (94) | **0.1 (0.0 - 0.8)** | **0.032** |
|  | T | 4 (33) | 2 (6) | 1 |  |
| rs1800629 - TNF-α_-308G>A (Regulatory) | | | | | |
|  | GG | 5 (83) | 15 (88) | N/A | 0.999 |
|  | AG | 1 (17) | 2 (12) |  |  |
|  | AA | 0 (0) | 0 (0) |  |  |
|  | G | 11 (92) | 32 (94) | 0.7 (0.1 - 8.3) | 1.000 |
|  | A | 1 (8) | 2 (6) | 1 |  |
| rs7958375 - CUX2_c.64-11987G>A (Intron) | | | | | |
|  | GG | 3 (50) | 13 (76) | N/A | 0.318 |
|  | GA | 3 (50) | 4 (24) |  |  |
|  | AA | 0 (0) | 0 (0) |  |  |
|  | G | 9 (75) | 30 (88) | 0.4 (0.1 - 2.1) | 0.355 |
|  | A | 3 (25) | 4 (12) | 1 |  |
| rs319952 - AGBL4_c.206+5387T>C (Intron) | | | | | |
|  | TT | 2 (33) | 9 (53) | N/A | 0.342 |
|  | CT | 4 (67) | 5 (29) |  |  |
|  | CC | 0 (0) | 3 (18) |  |  |
|  | T | 8 (67) | 23 (68) | 1.0 (0.2 - 3.8) | 1.000 |
|  | C | 4 (33) | 11 (32) | 1 |  |
| rs320003 - AGBL4_c.91+2046C>T (Intron) | | | | | |
|  | CC | 3 (50) | 11 (65) | N/A | 0.362 |
|  | CT | 3 (50) | 4 (24) |  |  |
|  | TT | 0 (0) | 2 (12) |  |  |
|  | C | 9 (75) | 26 (76) | 0.9 (0.2 – 4.2) | 1.000 |
|  | T | 3 (25) | 8 (24) | 1 |  |

N/A: Not applicable

ADME: Absorption, distribution, metabolism and excretion. The metabolism of drugs is divided into three phases including modification (phase I), conjugation (phase II), and excretion (phase III). n: the numbers of observed genotypes/alleles and corresponding percentage.
